# Non-Laboratory Project-Based Learning for Final Year Bioscience Students: Lessons From COVID-19

**DOI:** 10.3389/bjbs.2023.11561

**Published:** 2023-08-21

**Authors:** Declan J. McKenna

**Affiliations:** School of Biomedical Sciences, Ulster University, Coleraine, United Kingdom

**Keywords:** COVID-19, honours project, wet-lab, dry-lab, bioscience

## Abstract

**Background:** Provision of “dry-lab” final year honours projects, based outside the laboratory, have been proposed as a viable alternative to traditional “wet-lab” projects in bioscience subjects, but their value has not been widely evaluated to date. In 2020–21, the COVID-19 pandemic meant all students in the School of Biomedical Sciences at Ulster University (UU) undertook dry-lab projects, due to campus lockdown. Therefore, this provided an ideal opportunity to evaluate the provision of dry-lab projects in a large student cohort.

**Methods:** A pilot group of final year students (*n* = 4) studying Biomedical Science at UU were interviewed to evaluate their experience of conducting a dry-lab project. This evaluation and the themes that emerged were subsequently used to inform the co-creation of a survey to appraise student experience of dry-lab research project learning across the final year student cohort in School of Biomedical Sciences (*n* = 140). Quantitative and qualitative data was collected and analysed for trends and themes.

**Results:** The results of this project identified four main themes related to dry-lab projects; expectations, skills & employability, quality of experience and choice. Student expectations about dry-lab projects were not dramatically changed, although initial negative opinions of some individuals were over-turned. Most students recognised that they had developed many useful employability skills through dry-lab projects, although lack of practical laboratory experience was still perceived as a drawback. Student experience was influenced by personal circumstances but students reporting poor project experience had significantly lower levels of communication with supervisor (*p* < 0.05). Most students agreed that choice of dry- and wet-lab projects would be valuable for future cohorts.

**Conclusion:** This report concludes that dry-lab project provision can be a suitable and equitable alternative for wet-lab projects. Dry-lab projects can be valuable for learning new skills and may be an attractive option for some students and supervisors who prefer to work outside the laboratory setting. A choice of both dry-lab and wet-lab projects is highly recommended as it provides more choice for students to tailor their final year experience to their individual circumstances, strengths and future career aspirations.

## Introduction

In UK higher education, the final year honours project is a highly valued component of the university degree, representing a “gold-standard” stamp of academic excellence that provides students with important research skillsets for employment after graduation [1]. In Science, Technology, Engineering and Maths (STEM) subject areas, it is typical that project-based learning in the final year of a science degree is a laboratory-based experience (colloquially known as “wet-lab”). However, this traditional arrangement has been challenged in recent years, with many universities producing evidence to illustrate that students can benefit equally from “dry-lab” science projects based outside the laboratory [2, 3].

This is a welcome development which helps overturn the stereotypical view of the scientist [4]. Scientists in the workplace will spend much of their time writing, interpreting data, communicating science and working at computers, in addition to laboratory bench work. Since employability and workforce readiness are integrated concepts in many University science degrees, it is therefore appropriate that students have the option to develop these extra non-laboratory skills by providing a more diverse range of projects at final year, including dry-lab projects. Such projects could be data-analysis, computational projects, systematic reviews, meta-analyses or tailored presentations [5]. However, there is an understandable reluctance by educators to change from “tried-and-tested” laboratory-based approaches that have worked successfully in the past, not least because there is evidence that science students do not consider dry-lab projects to be as worthwhile as wet-lab experiences [6]. The challenge for our School of Biomedical Sciences at UU, similar to departments in other institutes, is to change preconceptions and expectations about dry-lab projects amongst both students and educators.

It is important this challenge is addressed, because various academic, economic and pedagogic factors mean that project-based learning practices must undergo significant revision to create a sustainable, inclusive model of final year research project provision for future cohorts of students. For example, as student numbers increase in UK higher education, there is increasing financial pressure on universities to provide relevant wet-lab projects in suitably equipped environments [7]. Hence, as student numbers increase, dry-lab projects are a more financially viable option and have the extra benefit of being more environmentally friendly. Moreover, providing a dry-lab project workflow will allow students to work through project activities in a virtual environment in their own time without the need for a supervisor in attendance and without having to access to laboratories facilities at scheduled times. Employed properly, the dry-lab project can therefore be more efficient in terms of organisation, time-commitment and availability of those involved [8]. Furthermore, increased student recruitment to distance-learning courses means that provision of dry-lab projects will need to become more common. Indeed, the importance of the “remote laboratory” in STEM-related education has been identified as a key resource in promoting internationalization, as well as access to education for traditionally underrepresented groups [9]. This will also be attractive to students who find wet-lab project provision problematic because of circumstances that make it difficult for them to attend laboratories in person. Dry-lab projects may well be a more attractive option for students in this position and would address inclusivity and accessibility issues in the process [10]. Importantly, this also has value beyond Biomedical Sciences, since many other science disciplines are facing similar pressures, so case studies of successful dry-lab projects will be very important for inspiring and motivating colleagues to develop their own projects [3].

For change to happen, however, evaluation is needed to demonstrate the value of this type of project. This is very achievable, since dry-lab research projects in science is not a new idea [7]. Indeed, there are many examples of free and commercial resources that are available to educators to help virtual teaching [11]. However, there is still a need to robustly evidence that such approaches are fit-for-purpose, both in terms of pedagogy and value to stakeholders. At UU, the disruption caused by the COVID-19 pandemic in 2020 ended up providing an ideal opportunity to do this, since all students in the School of Biomedical Sciences in the academic year 2020–21 undertook dry-lab projects as laboratory access was prohibited. Therefore, this review describes a project that was developed to evaluate alternative options to traditional laboratory-based projects.

## Methods

### Participants

A pilot group of four final year students studying BSc (Hons) Biomedical Science were randomly assigned to complete a dry-lab research project under the supervision of principal investigator (PI). The students and PI collaborated to apply their personal experience of this type of project-based learning to the co-development of a survey which would subsequently evaluate the student experience of dry-lab research project learning across the entire final year cohort in School of Biomedical Sciences. The pilot group took part in focus groups and the themes identified were embedded into the survey design exploring the student experience of dry-lab project-based learning. The survey was released to all final year students in the School of Biomedical Sciences, who were all undertaking some form of dry-lab research project. The data collected from this survey (*n* = 140 respondents) provided important quantitative and qualitative baseline data from the year group for further evaluation.

### Ethics

Ethical implications for the project were also considered carefully and approval granted by Centre for Higher Education Research and Practice (CHERP) at UU (Ref:CHERP-20-001). Students in the pilot group were provided with a Participant Information sheet about the project, explaining (i) how their feedback may be used and (ii) that their decision to partake (or not) will have no impact on the support they receive during their project. For the wider survey of the entire final year student cohort, students were again under no obligation to complete the questionnaire. Completion of questionnaire implied consent, but no student was penalised for opting out. All responses were anonymised and responses collected were held confidentially by the primary researcher under password protected access.

### Evaluation Design and Justification

Evaluation for this project was based upon scrutiny of the quantitative and qualitative data collected via the tools and approaches below. Throughout, the project aimed to align with guidance provided through the UU’s Strategy for Learning and Teaching Enhancement (SLaTE) [12].

Peer-led focus groups were held to evaluate and discuss project direction. This type of collaborative learning was deemed appropriate for students to become actively involved in shaping education experience for their peers [13]. Indeed student-staff partnerships are an increasingly important part of Higher Education, offering much scope for innovative pedagogic practice [14]. The student input is integral to changes in curriculum, and this type of partnership helps the case for students as agents of change [15]. Importantly, in terms of manging power imbalance and possible bias, students were made aware that these focus groups were not linked to the assessment of their work. Instead, they understood that they were invited to collaborate in co-developing the nature of the survey for the wider cohort of students, by recommending questions/themes that would provide information they would like to know.

Semi-structured interviews were employed as a qualitative research method which can explore deeper opinions about a given topic [16]. In this project, semi-structured interviews were conducted with the sub-group of four students. To avoid possibility of power bias, these were conducted by a colleague of the PI. There are drawbacks to semi-structured interviews, since they are time-consuming and it is difficult to canvas large numbers, so there may not be sufficient data to inform meaningful analysis [17]. Nevertheless, they were very suitable for this project as one element of a mixed methods data collection, from which themes could be extracted and explored further in a larger cohort via a bespoke survey.

Student surveys are a long-accepted method for collecting feedback from students on education experience. However, they must be properly designed and conducted to ensure useful data is collected [18]. That is why it was important that the finalised set of survey questions for this project was informed by the focus-groups mentioned above and by informal discussions with colleagues. Survey responses provided information on how students felt about the project-based learning, skills accrued, support they received and how they feel they met learning outcomes, in order to provide a rich source of qualitative and quantitative data to robustly evaluate student experience of dry-lab projects.

### Data Collection and Analysis Procedures

Data collection was managed using REDCap electronic data capture tools hosted at UU. REDCap (Research Electronic Data Capture) is a secure, web-based software platform designed to support data capture for research studies [19]. It provides 1) an intuitive interface for validated data capture; 2) audit trails for tracking data manipulation and export procedures; 3) automated export procedures for seamless data downloads to common statistical packages; and 4) procedures for data integration and interoperability with external sources.

Results were a ‘mixed methods’ combination of qualitative and quantitative data, gathered from the methodologies listed above. Together these combine to inform a grounded theory approach to the project [20]. Rather than being entirely linear, this allowed for some flexibility, adapting approaches in response to both the data collected and surrounding discussions. Quantitative data was provided through the ‘scored’ questions on the bespoke survey issued to students (i.e., where a rating is selected against a question). This data was presented to allow comparisons of answers from linked questions where appropriate. This helped visualise any changes in responses which might have occurred by the experience of undertaking a dry-lab project. Statistical significance was assessed by paired *t*-test with data considered significant where *p < 0.05, **p < 0.01, ***p < 0.001. Qualitative data was collected from the focus group, semi-structured interview and from open questions on the bespoke survey. This data was reviewed and analysed for thematic content by a six-step process [21, 22]. The grounded theory approach helped evaluate how the findings can be used to potentially inform further data analysis in future. The final structure of the reporting was informed by guidance about aligning outcomes with objectives [23].

### Reflective Practice

The collection and analysis of data was informed throughout this project by collegial discussions with colleagues, including course directors, final year project module coordinators and other colleagues. To capture the formative ideas that arose from these discussions, and the journey of the primary researcher through the process, a reflective journal of notes was kept throughout the process [24]. This captured evolving perceptions, project progress, key decisions and personal reflections on the transformative experience of doing the project and learning new research approaches (particularly analysis of qualitative data). This reflection in turn influenced the critical thinking within the discussion below.

## Results and Discussion

### Pilot Group Results

The pilot group of students (*n* = 4) in this project were placed in the subject area of genetic medicine, therefore bioinformatic analysis was adopted as the basis for a dry-lab project, since it aligns with wider advances in cancer research and analyses of health-related patient data [25, 26]. A project was therefore designed which would substitute traditional wet-lab activities with computer-based ones, while remaining focused on the area of genetic medicine.

The value of this approach was then evaluated through focus groups and semi-structured interviews, which would be used to inform and co-develop the survey for canvassing the experience of the wider student cohort. This idea of student as partner or ‘producer’ encourages collaborative relations between student and academic to generate knowledge [27]. The analysis of this information revealed four main themes, which are summarised and itemised in Table 1 below.

**TABLE 1:** Summary of responses collected from focus groups and semi-structured interview(s) with Pilot student group, including identification of key themes.

Area of discussion	Summary of responses	Theme identified
Defining dry-lab science projects (prior knowledge, experience & expectations)	• Didn’t know what to expect in general	Expectations
	• Had heard term “dry-lab,” did not know what it meant	
	• Won’t get the same skill as in wet lab	
	• Not the skills needed for job	
	• No chance to develop new techniques	
	• Wet lab experience can help understand the work better (learning by doing)	
	• Wouldn’t be in the lab, all computer based	
	• Might be disadvantaged to other years, may not get the same experience in comparison to wet lab	
	• More flexibility (time and travel)	
Expertise Gained (skills, transferable knowledge)	• learned more skills (bioinformatics, online and computer skills) than were not expected	Skills & Employability
	• Been looking at job applications and they are asking for IT skills	
	• As they are new and transferable skills, I believe I have more to put on the table in a job	
	• Employers are looking for bioinformatic skills	
Advantages & Disadvantages	• Surprised on how different the experience was compared to expectation	Student Experience
	• A lot less stressful than expected	
	• Things ran smoother than expected, got results easier without much waiting	
	• Some challenges in communications as it relied on emailing back and forth rather than being beside someone to point things out	
	• Been enjoyable, flexible	
	• No standing around	
	• It worked a lot better than expected	
Recommendations	• would be happy doing dry lab again	Choice
	• Yes if the supervisor is like is good at responding to emails and communication; no if the supervisor is not a good communicator	
	• Depends on supervisor	
	• Depends on flexibility for individual student	

These reveal that students really did not know what to expect about a dry-lab project, principally because they had little exposure to, or awareness of, what it might constitute. One student statement summed up the apprehension in the pilot group about studying outside a laboratory;

“Prior to starting my project, I was sceptical as to how a dry lab project would be carried out and if it would be just as beneficial as doing a wet lab-based project.”

In terms of skills, there was a fear that lack of laboratory skills would be a drawback, although once the project progressed, students became more aware of the variety of skills being accrued, including digital skills which employers might particularly value, as articulated by one student;

“Throughout my project I have learnt so many new skills that I did not expect to learn while doing a dry lab project. I believe I have a good understanding of bioinformatics and also really have improved my IT skills, this is so beneficial when applying for jobs as I have found these skills to be very important to employers.”

Student experience naturally included some negative and positive aspects, although there was a general acceptance in this small group that there was increased flexibility and less stress than expected;

“A benefit of this being a ‘dry-lab’ project was the flexibility around planning time for this project, studying for other modules and my part-time job.”

Perhaps most tellingly, there was a general consensus that preconceptions about dry-lab projects had been somewhat over-turned, with acknowledgement that it would be acceptable choice in future;

“I used to think I was a very hands on learner and would not be able to learn anything from a computer screen rather than real life however this year has definitely changed my opinion of online learning and wet lab projects.”

“I do not feel disadvantaged using this experience versus a ‘wet-lab’ final year project experience. I would definitely recommend a ‘dry-lab’ based project to others.”

However, it was pointed out that the role of the supervisor, especially in communicating effectively throughout, would be paramount in ensuring a good overall experience. The themes identified in Table 1 were then discussed further with the pilot group of students to co-develop the survey design for further exploration and validation. The focus was on asking questions which would determine if the experiences and opinions of the pilot group were matched across the entire cohort of final year students. As a result, the final survey was designed to incorporate four sections, each aligned with a different theme as shown in Table 1. The survey was released to all final year students and the data collected is presented and discussed below.

### Overall Student Survey Results

**FIGURE 1:**
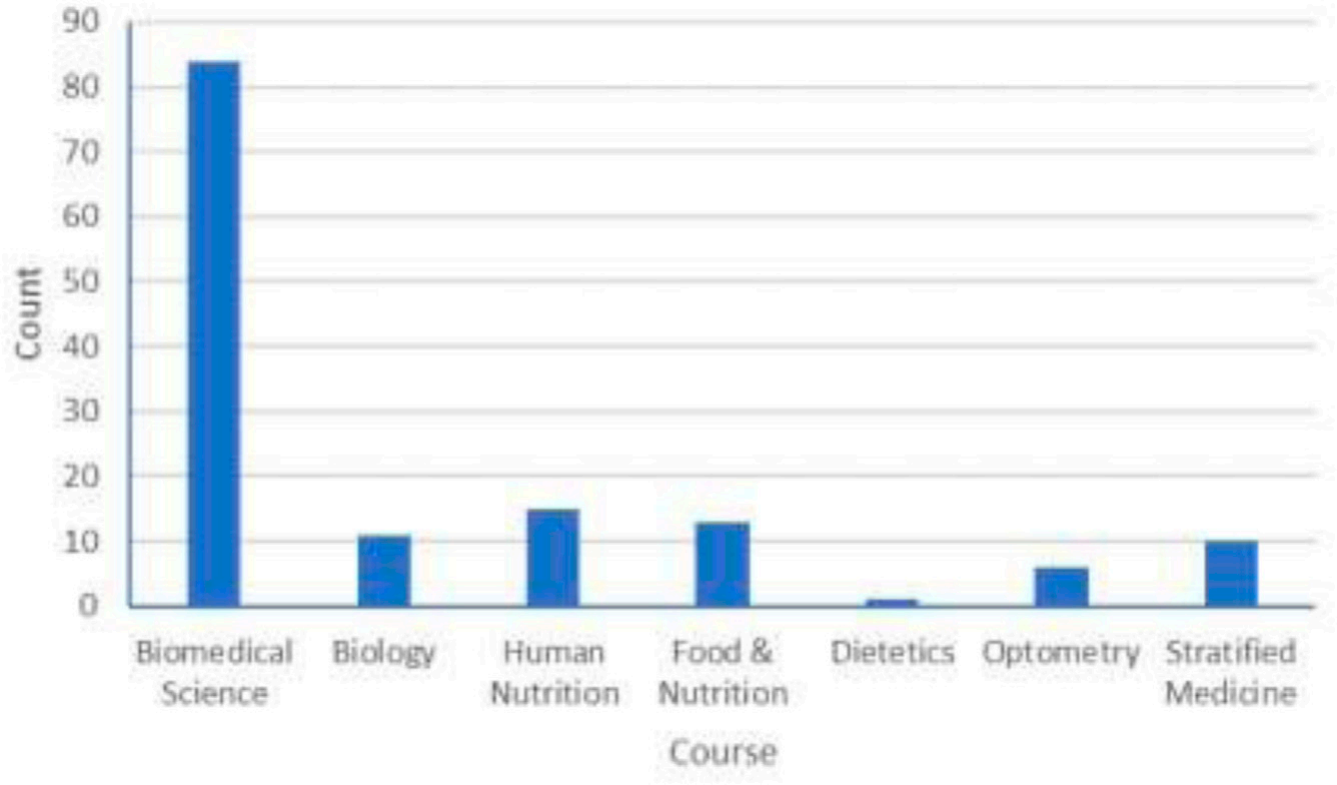
Number of respondents from each of the seven courses in School of Biomedical Sciences. [Data: (No. of responses, % of survey respondents): Biomedical Science (84, 60.0%), Biology (11, 7.9%), Human Nutrition (15, 10.7%), Food & Nutrition (13, 9.3%), Dietetics (1, 0.7%), Optometry (6, 4.3%), Stratified Medicine (10, 7.1%)].

The survey results were collected and analysed for both overall trends and thematic content. A total of 283 final year students from 7 different courses were contacted on 3 occasions over a 3-week period following completion of their projects in May 2021. 140 responses (49.5%) were received, primarily from the Biomedical Science course, which was not unexpected as it consists of 3 separate cohorts and has about four times as many students as each of the other courses (Figure 1).

The data collected from the other questions in the survey were analysed and have been presented below against the four themes identified in Table 1 for ease of understanding and discussion.

#### Expectations

**FIGURE 2:**
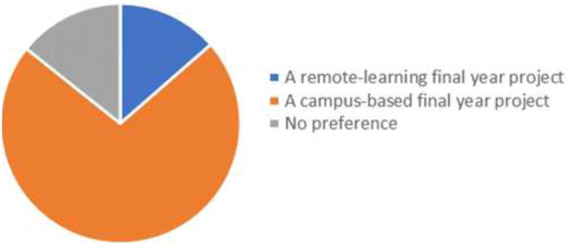
Comparison of student preference for remote-learning (dry-lab) or campus-based (wet-lab) final year project. [Data: A remote-learning final year project (19, 13.6%), A campus-based final year project (101, 72.1%), No preference (20, 14.3%)].

It was not surprising to learn that a substantial number of students preferred to do a wet-lab one when they were asked to reflect on their preconceptions about dry-lab projects at the start of the year (Figure 2). This confirmed data from elsewhere which found similar attitudes among students [6, 28]. Although this question depended on students recalling how they felt several months before survey was completed, it is still likely to be a true reflection of the apprehension about dry-lab projects which was also apparent in the pilot group of students. This is linked to a lack of knowledge about what constitutes a dry-lab project, which is understandable since exposure to this type of project is limited in undergraduate degrees [3]. The more pertinent question was whether the experience of undertaking a dry-lab project would change that preconception.

**FIGURE 3:**
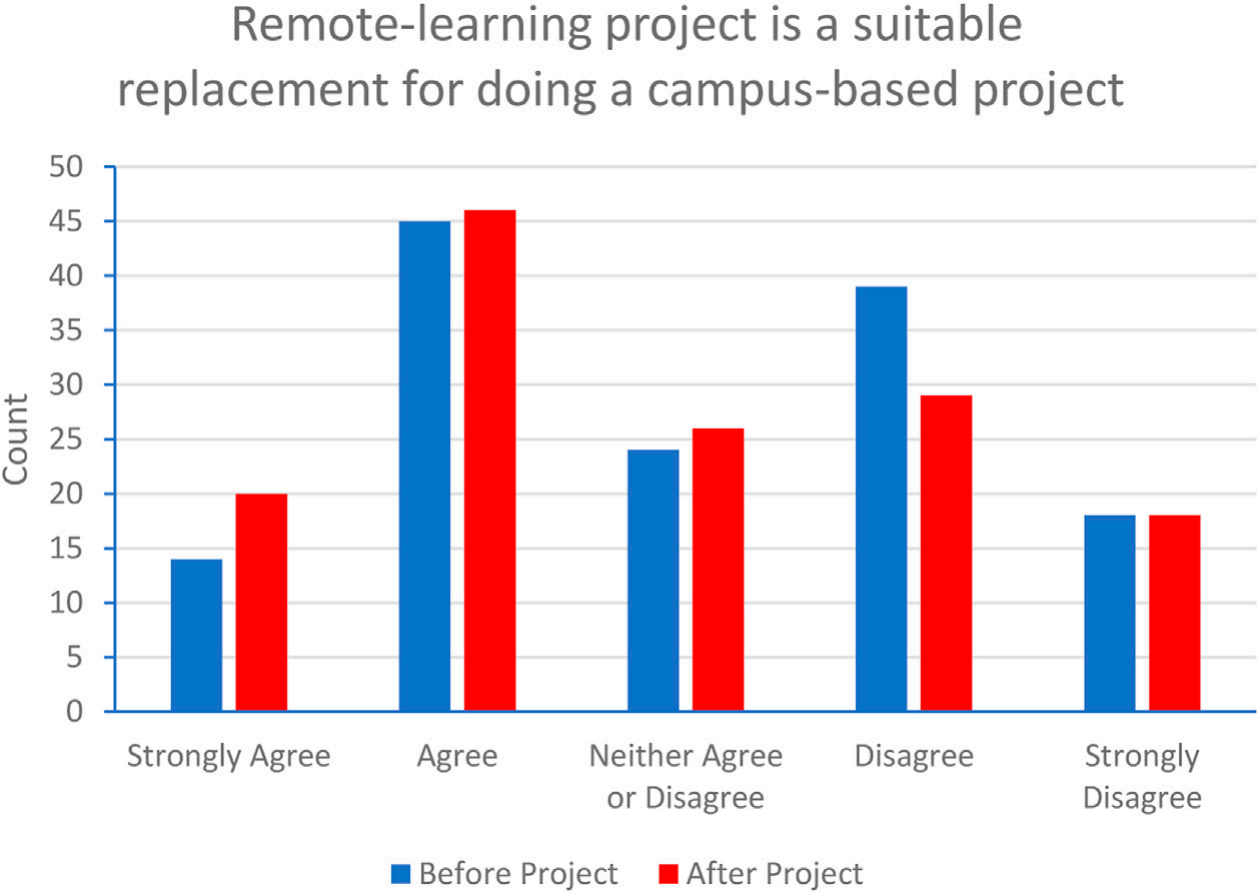
Student opinion, before and after project completion, on whether a remote-based project was suitable replacement for campus-based project.

To explore this further, the students were asked if they were satisfied that doing a remote-learning project was a suitable replacement for doing a campus-based project, but were also challenged to consider if their opinion had changed from initial expectations by the end of the project. Figure 3 shows a comparison between the answers before their individual project began and after it was completed.

Again, this question depended on students recalling how they felt at the start of the project so we must be cautious about the interpretation of this data. The graph only shows overall numbers and does not compare how individual students voted before and after project completion. However, more nuanced information can be found in the analysis of the qualitative data about how expectations were challenged and, in some cases, overturned. The reflective quotes from one student illustrates how the experience of dry-lab projects changed their opinion;

“Before starting the investigative project, I was concerned about it being a dry lab experiment. I wanted to get the best grade possible and was not sure this was going to be possible without being in a lab doing the experimental work myself as well as having the supervisor present.”

“Now that I have completed my project I would recommend a dry lab project to everyone after understanding all the skills I have gained this past academic year that I would not have been able to gain while doing a wet lab experiment.”

**FIGURE 4:**
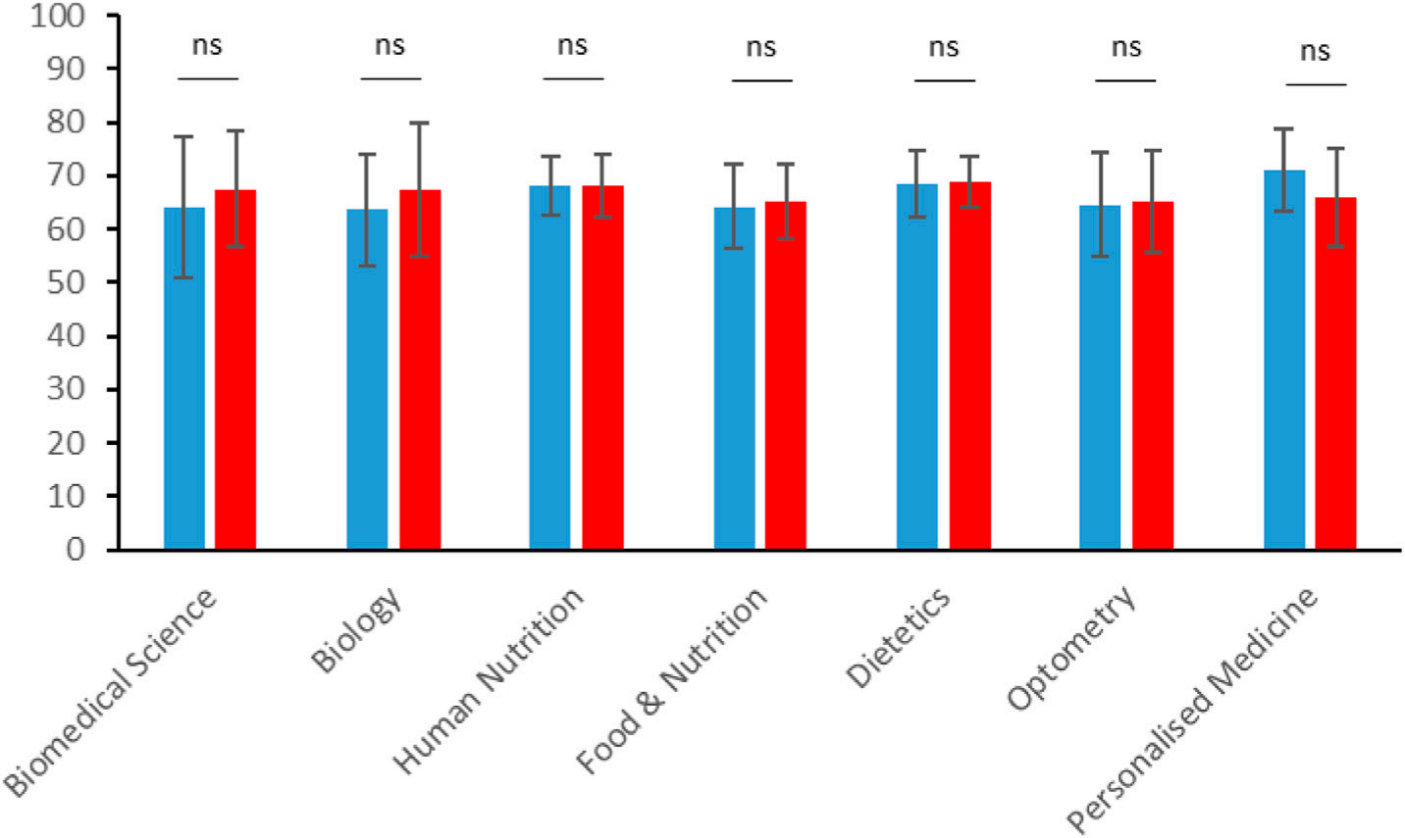
Average scores for dry-lab projects in 2020–21 (red bars) were not significantly different from average scores for wet-lab projects provided in 2019–20 (blue bars) in any of the courses (Data shown is Mean ± SD. Student’s t-test; ns, non-significant).

Importantly, the dry-lab project provision did not significantly impact on the average marks for each of the 7 courses across the School (Figure 4). This demonstrates that the learning outcomes for the final year project modules can be met by dry-lab project provision and that students do not experience a grading advantage or disadvantage from this type of project. This is important if a choice of dry- and wet-lab projects are to be offered together in future, so one option is not seen as academically “easier.”

#### Skills and Employability

**FIGURE 5:**
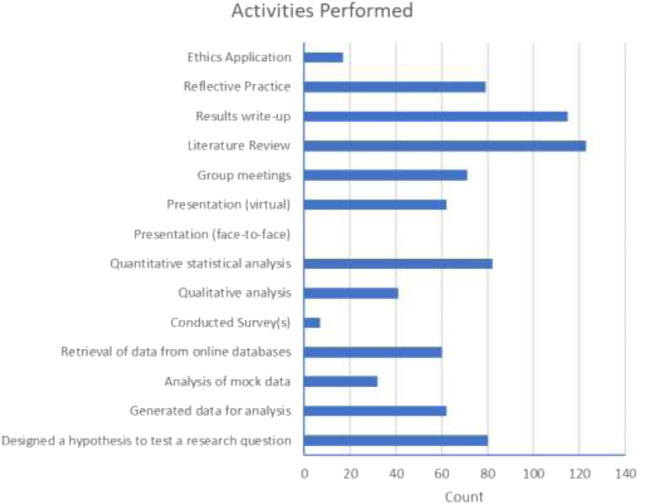
Activities identified by students as having been undertaken in their dry-lab projects.

The pilot group of students felt it was important that the survey gave their peers the opportunity to identify and confirm what activities they performed during their project, so that they could appreciate the scientific skills they were accruing. As Figure 5 below shows, students undertook a wide range of different methodologies, analyses and presentation approaches across the various dry-lab projects. This emphasises that laboratory activities constitute only one element of science projects, so dry-lab projects can effectively provide experiences in many other important science skills.

**FIGURE 6:**
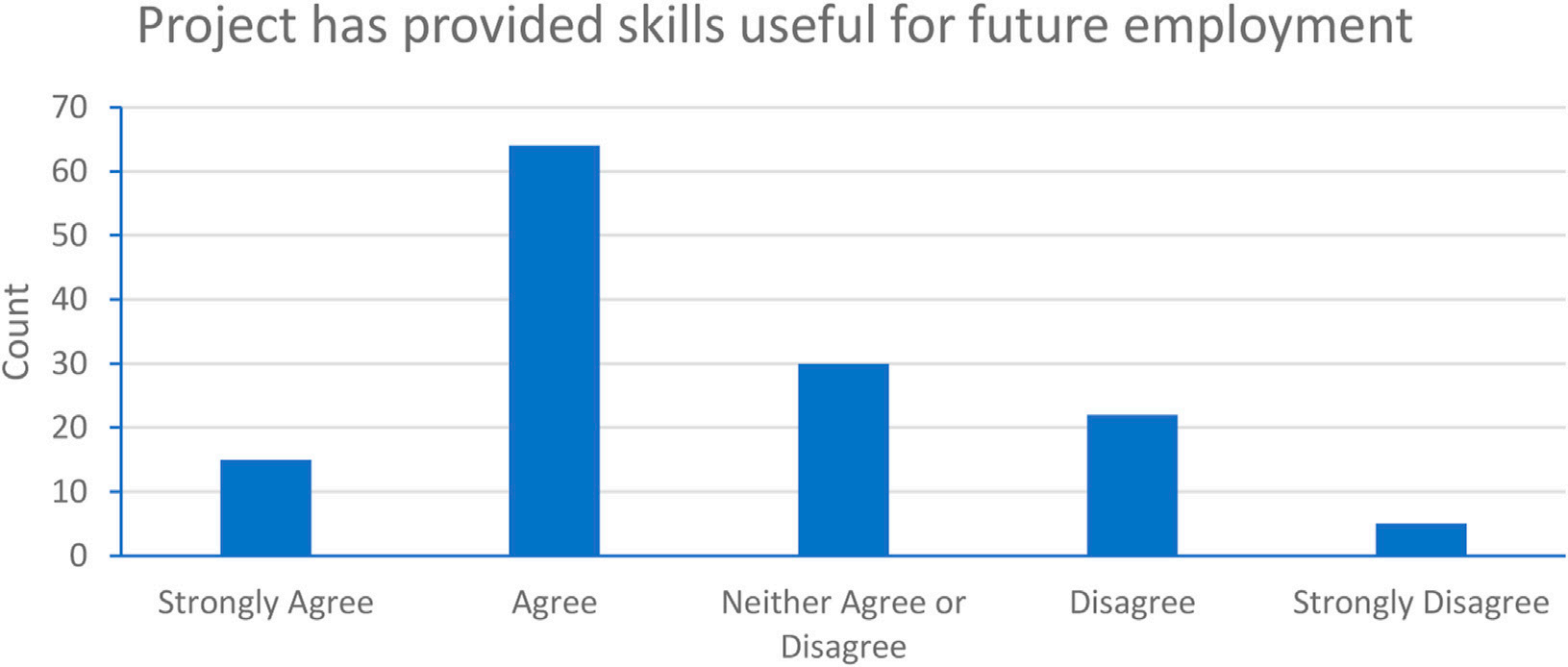
Student opinion on whether dry-lab projects would be useful for employability. The majority of students either strongly agreed (15, 11.0%) or agreed (64, 47.1%) that this was the case. [Other Data: Neither Agree or disagree (30, 22.1%), Disagree (22, 16.2%), Strongly disagree (5, 3.7%)].

This type of data is valuable because it allows educators to demonstrate to students that these are skills that employers in the Life Sciences sector (and beyond) value in graduates and prospective employees [29]. Encouragingly, the majority of students (58.1%) did recognise that their dry-lab project experience had provided them with skills that will be useful in future employment (Figure 6).

This is important for students to realise and to be able to articulate in future job applications and interviews, since work-based projects in the Life Sciences sector will involve a blend of hands-on practical skills with digital literacy and computational acumen [30]. In a typical bioscience degree, most students will already have significant wet-lab practical skills from modules completed in Year 1 and Year 2 of their degree, while a significant proportion of them will also have gained working laboratory experience in their placement year. What they may not have gained is exposure to the non-laboratory skills which are equally important in science-related jobs. Dry-lab projects therefore offer the chance for students to complement their laboratory experimental skills with digital experimental skills [31]. In this cohort of respondents, there did appear to be general acknowledgement of this fact.

**FIGURE 7:**
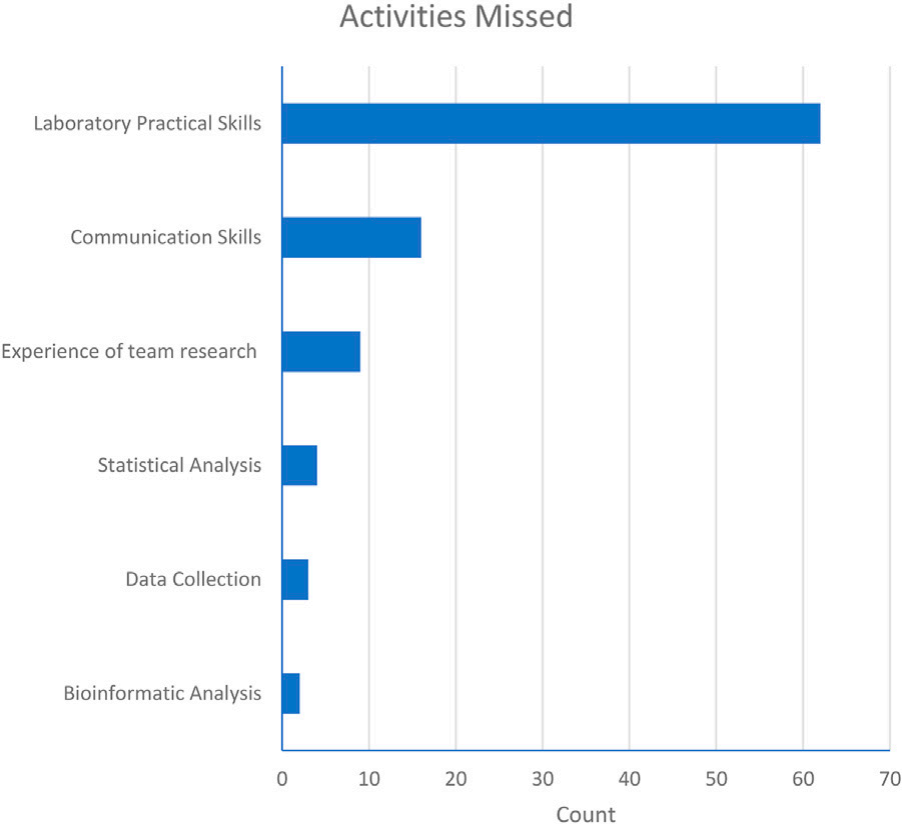
Activities not experienced by students during dry-lab projects indicating clearly that lack of practical laboratory experiences was undoubtedly recognised as a deficit.

However, dry-lab projects simply cannot substitute every aspect of the wet-lab experience, so it was not surprising to find that students clearly recognised they had missed out on exposure to laboratory skills at Level 6 (Figure 7).

It therefore seems sensible that the optimal final year project would have a blended approach, allowing students to get both hands-on laboratory exposure and digital familiarity so they can build a broad base of demonstrable skillsets. Key to this is variety and choice of project, which is discussed further below.

#### Experience

**FIGURE 8:**
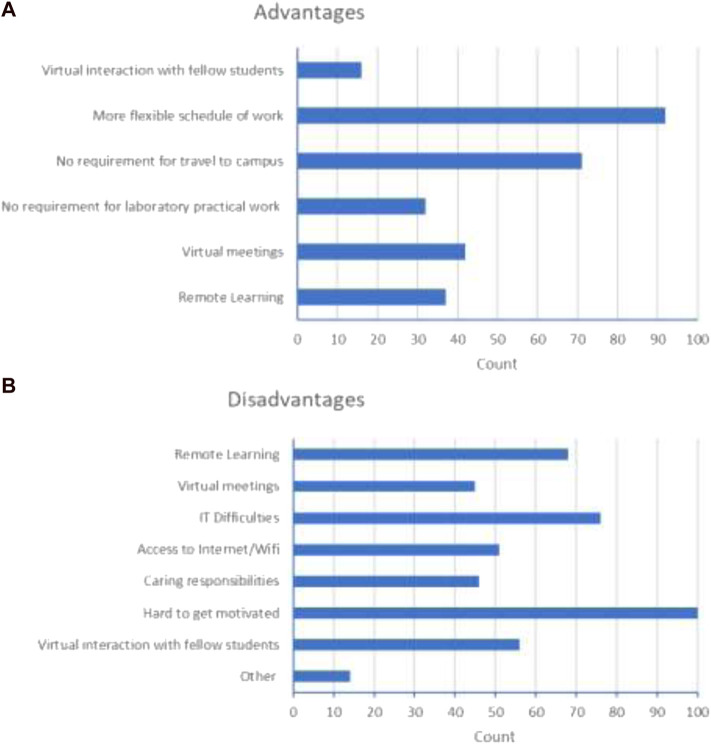
Elements of the dry-lab project experience which students considered to be **(A)** advantages and **(B)** disadvantages.

The student experience of dry-lab projects was captured in terms of elements which students identified as being advantages or disadvantages (Figures 8A, B). Of course, the wider context of the pandemic is an important factor to consider in reviewing this data, but it should still provide some insight into the aspects of dry-lab project provision which students found beneficial or not.

Interestingly, elements which some students considered appealing were considered drawbacks by other students, illustrated by the two contrasting quotes below.

**Positive:** “We had flexibility of working times, additional time available by not having to travel to campus and plenty of support.”

**Negative:** “Some students may find it useful to work from home but personally I felt at a huge disadvantage. I struggle to work from home and concentrate.”

This again emphasises the diversity which exists within the student cohort in terms of personalities, preferences, responsibilities and requirements. It therefore follows that improved variety and choice of final year projects will be welcomed by students who want a project which best fits their personal circumstances.

**FIGURE 9:**
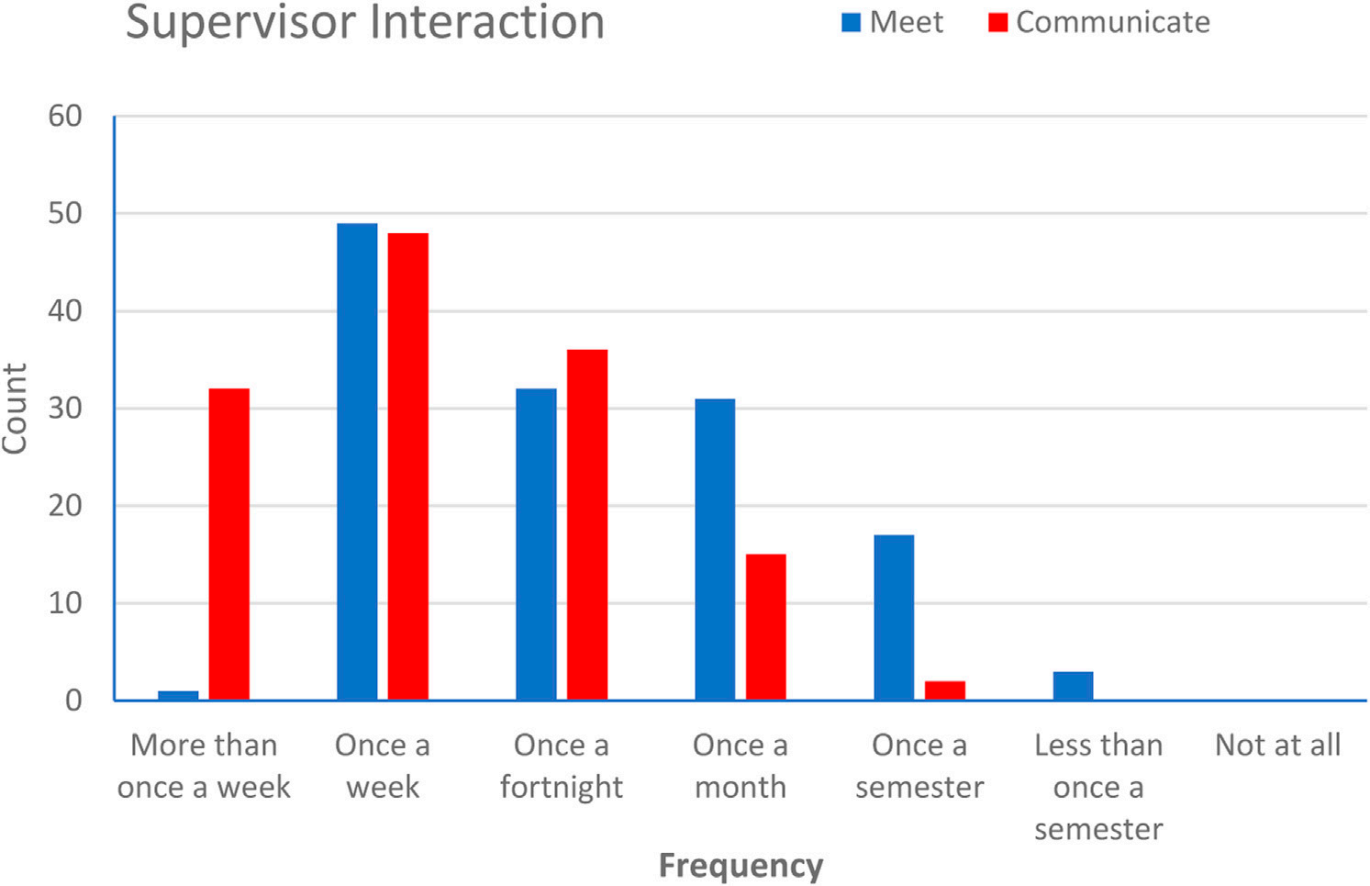
Frequency of supervisor interaction with student via meeting (live virtual, synchronous) or communication (phone, email, asynchronous).

However, one key aspect (not explicitly shown in Figure 8 above) which shaped the experience of the dry-lab project was linked to the relationship between student and supervisor. Whilst this has always been the case for any research project [32], it appears to be even more essential when the communication is primarily through virtual means, as it has been for the past year. Figure 9 below shows the data collected for contact frequency and type of contact between student and supervisor. “Meet” indicated synchronous meetings, typically by virtual tools such as Zoom. “Communicate” mostly referred to asynchronous contact, such as email.

**FIGURE 10:**
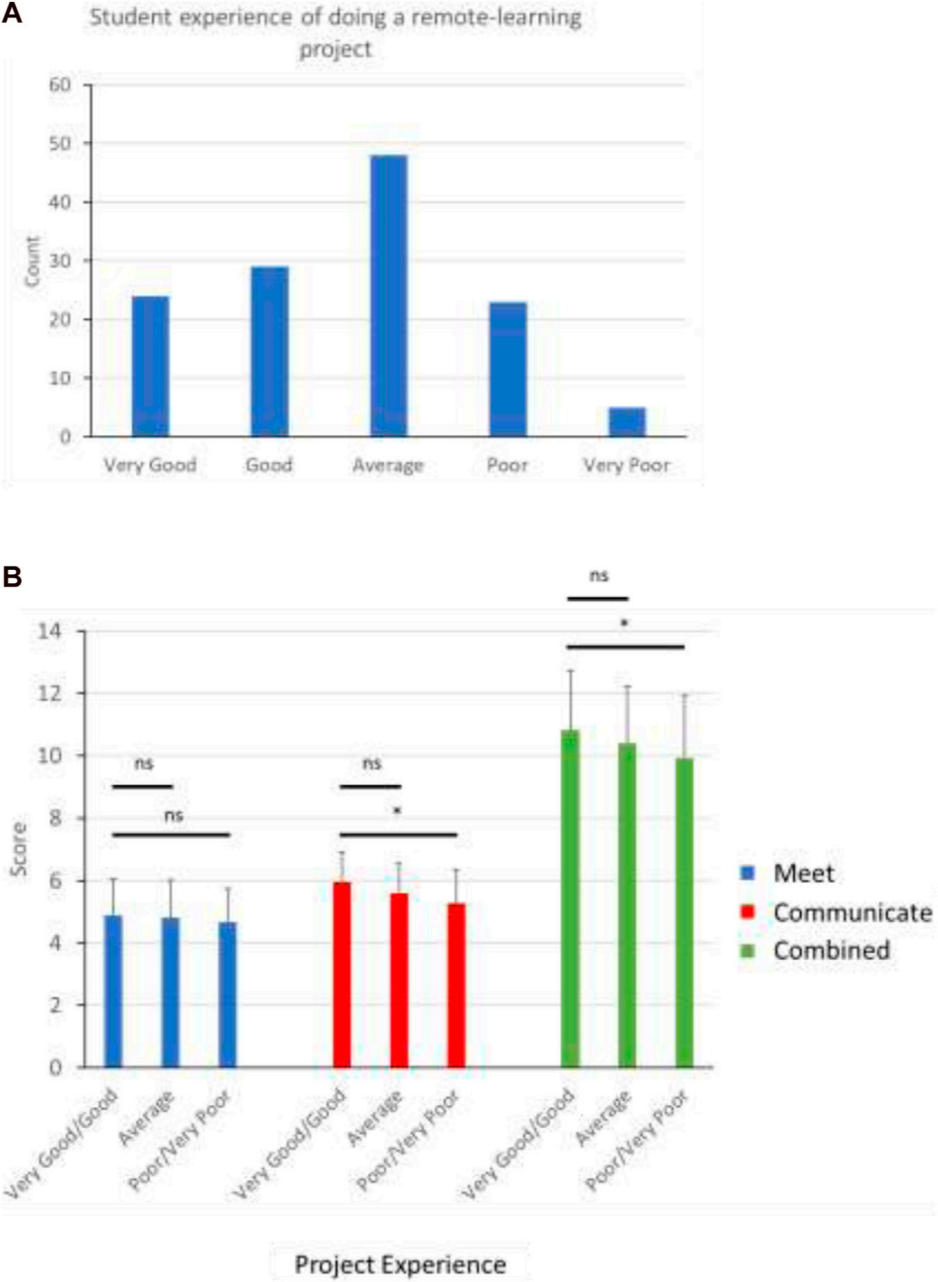
**(A)** Overall experience of student respondents undertaking dry-lab project provision in School of Biomedical Sciences 2020–2021 **(B)** Students who reported a very good or good experience had a significantly higher level of communication with supervisor than those reporting a poor or very poor experience. [Data shown is Mean ± SD. Scores based on student reporting of average interaction with supervisor during project; 7 = More than once a week, 6 = Once a week, 5 = Once a fortnight, 4 = Once a month, 3 = Once a semester, 2 = Less than once a semester, 1 = Not at all (Student’s t-test *p*-values; *p < 0.05, ns = non-significant)].

Regardless of the type of contact, the frequency of communication was very important in making sure students felt supported and guided through their project. This is even more important in dry-lab projects, since students on-campus will usually have interactions in person with other laboratory members and researchers besides their supervisor. In home-based dry-lab projects, they are more reliant on supervisor alone, with even the normal interactions with fellow students more limited than usual. It follows that student who had less overall communication with supervisors were the ones who reported a poor or very poor experience (Figure 10).

The importance of supervisor interaction was captured succinctly by one student, who commented;

“Having a fantastic supervisor [meant] it was easy to keep organised with the workload ahead and what was involved in each part. However, I have known individuals who were not so lucky and their supervisor rarely contacted them and so they struggled. I believe this year your supervisor had a significant impact on your grade as you had very little interactions with other members of staff or students to talk through the project and how to approach it.”

#### Choice

**FIGURE 11:**
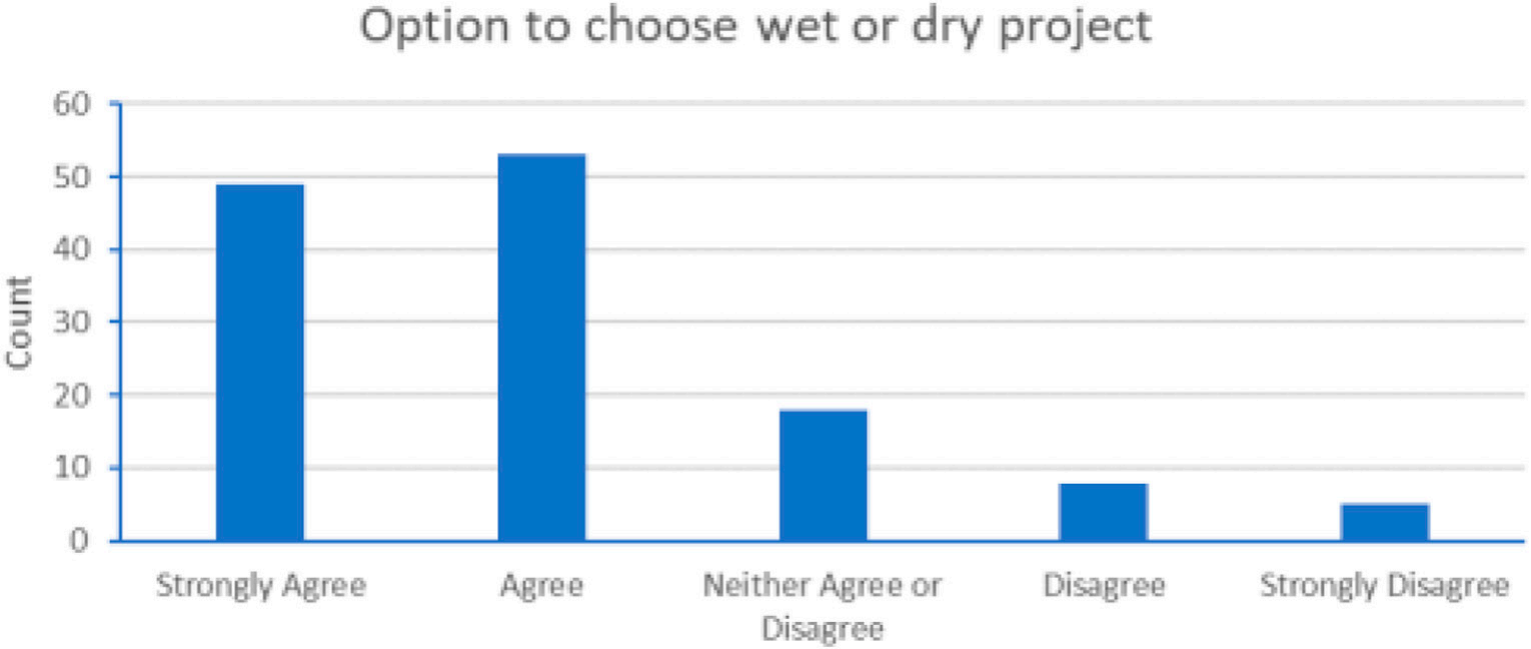
Students strongly agreed (49, 36.8%) or agreed (53, 39.8%) that a choice of wet- or dry-lab project should be made available for students in School of Biomedical Sciences. [Other Data: Neither Agree or disagree (18, 13.5%), Disagree (8, 6.0%), Strongly disagree (5, 3.8%)].

Despite the range of experiences that students recorded, and the various pros and cons identified in the process, the vast majority of students did think final year students in School of Biomedical Sciences should have a choice of wet-lab and dry-lab projects (Figure 11).

It is important to listen to this type of feedback from students. Offering an expanded range of final year project types affords students more choice to address gaps in their skillsets, thereby empowering them to improve themselves in accordance with the Student Learning Principles model outline in the current Learning & Teaching Strategy at UU [12]. Moreover, this type of project may be particularly attractive to students who may have personal circumstances which make attending wet-lab sessions difficult [33, 34]. As a School and course team, we are committed to supporting student wellbeing, which includes making recommended adjustments for students who may be experiencing difficulty with their allocated research projects for various personal reasons. In previous years, completion of wet-lab projects has been difficult for these students, due to circumstances with prevent them attending the laboratory sessions in person. A dry-lab project may well be a more attractive option for students in this position, rather than having to make ad-hoc adjustments to a wet-lab project to fit their needs [7]. This is illustrated nicely in this survey by one student, for whom dry-lab provision has been a very welcome development;

“I am autistic and while I did feel isolated this year due to remote learning, it was also much more comfortable being able to manage my own schedule with breaks to desensitize (an option that was not really possible during lectures and lab sessions for my entire course). Having a choice between a labwork-based project and a data analysis-based project is a great opportunity for the university to improve accessibility for neurodivergent students through accommodating and supporting them in a situation that suits their strengths and need.”

The quantitative data above is also supported by qualitative data, gathered from open-ended questions in the survey where students were given the opportunity to provide any other comments. A representative selection of these comments, both positive and negative, are shown in Table 2 below, again aligned against the four themes identified.

**TABLE 2:** Selected comments from the student feedback section of the survey.

Theme	Good comment	Critical comment
Expectations	“Although I had reservations at first I thoroughly enjoyed conducting my research project.”	“Remote learning is a poor replacement, more should have been done by the university to make it safe for students to be on campus”
	“Although wary at first I found researching and retrieving data as easy at home”	“I thought it would be a disaster”
Skills & Employability	“It allowed me to develop computer based skills that I otherwise would have lacked” “The remote learning project has actually allowed me to strengthen and deepen my statistical and critical analysis skills”	“Remote learning has prevented students from gaining vital practical skills to make them employable” “I would be completely unqualified to go and work in a lab environment now” “My remote learning project gave me no opportunity to gain new skills”
Student Experience	“Overall, due to the weekly communication between myself and my supervisor it really helped to resolve any problems I was having” “I enjoyed my online project and worked well with my supervisor as they were good at communicating with me.” “I believe a good motivated supervisor is key to working virtually.”	“Connection issues did make these meetings difficult on occasion” “It was difficult to get in contact with my supervisor and they rarely replied to emails.” “I needed and use facilities on campus as my own facilities for WiFi/technology are not very good” “I struggled a lot to get motivated”
Choice	“I think a few things could be improved- but it should definitely be an option post COVID”	“Remote-learning was okay. But it could never replace the social on-campus learning”
	“The opportunity for multiple types of data analysis needs to be offered for the student to have any benefit.”	“Unsatisfied with the delivery of final year projects during the pandemic and if given the choice I would not have chosen [sic] to go through this again.”
	“I would encourage the practice of remote learning to become common place in the future education in Ulster University.”	“If dry projects are offered in the future supervisors need to be well equipped and willing to work around those students.”
Overall	“Overall it was a very enjoyable experience.”	“The remote learning experience was an awfully challenging experience in my opinion”

**FIGURE 12:**
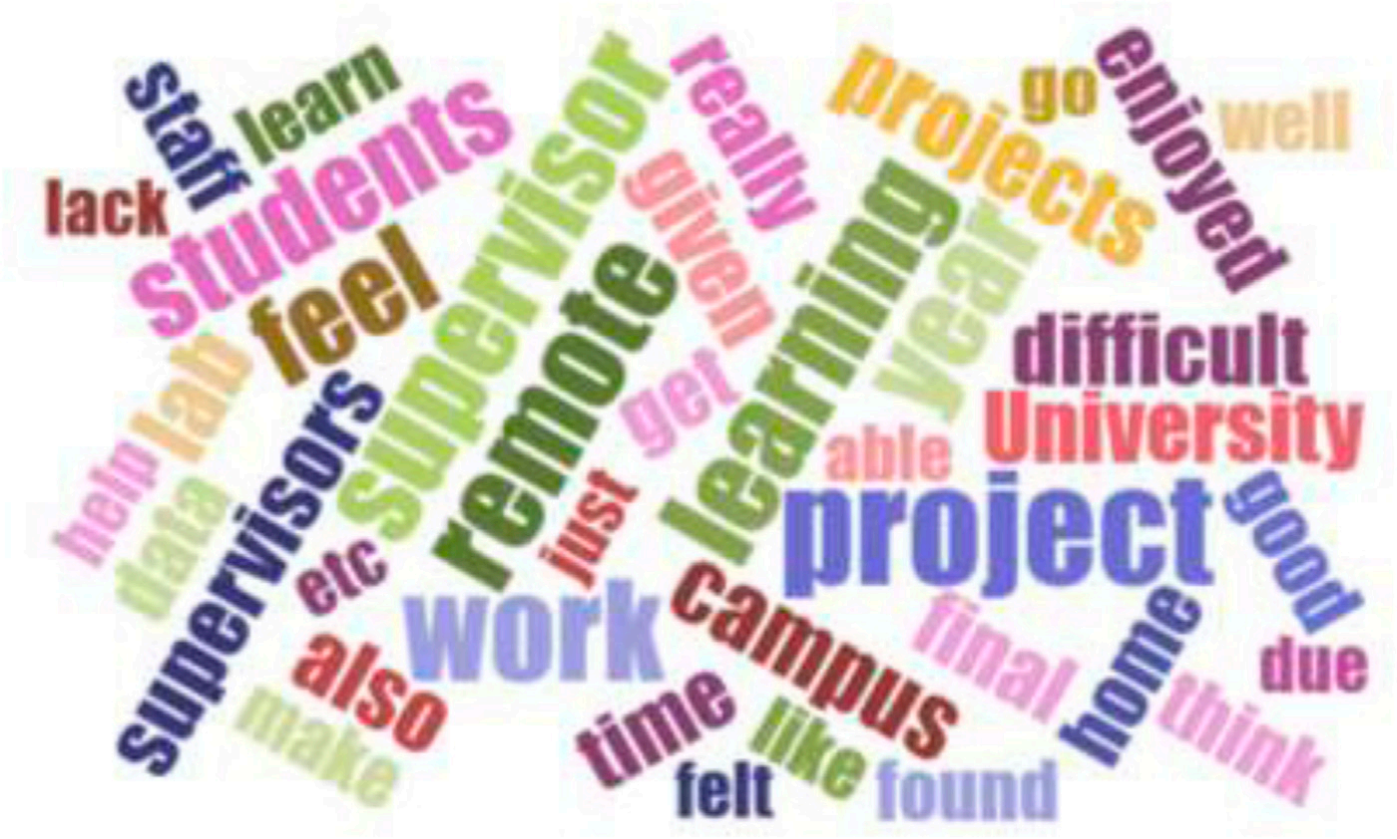
Word cloud generated form the text included in the comments provided by students in the open-ended feedback question of the survey, clearly indicating importance of supervisor(s).

Mirroring the data collected on student experience, the importance of the supervisor in the project was further evidenced by a word frequency analysis of these qualitative comments, as visualised in a word cloud (Figure 12).

Notably, the words “supervisor” and “supervisors” appear prominently, reflecting the frequency with which students mentioned how their supervision had contributed to either a positive or negative learning experience. However, it cannot be said for sure that this range is unique to the dry-lab project experience, since we do not have a similar set of data collected for student doing mostly wet-lab projects. Indeed, it is likely that the same variety in experience and a similar emphasis on good student-supervisor interaction would be reported for any cohort of final year students undertaking research projects. Other studies have similarly found that students associate research-focused staff with being less interested in teaching and in spending a reduced amount of time with their students [35]. This may create a tension between staff and student expectations, so it is important that supervisors understand their role may be different from that associated with traditional wet-lab projects. The data presented here reflects findings from other studies that show the challenge for improving student experience lies both in the provision of choice, allowing students to select projects that suit them [28], and in ensuring there is sufficient quality communication between supervisor and student throughout the project [36].

### Impact of Project for Academic Colleagues

The study aimed to demonstrate how colleagues could potentially address challenges that currently exist in the traditional model of final year science project provision. The impact upon colleagues at UU and in other academic institutes is likely to be improved by demonstrating the benefits in terms of finance, widening participation and workload.

For example, as student numbers increase in UK higher education, there is increasing economic pressure associated with providing relevant wet-lab projects in suitably-equipped environments [7]. This problem is exemplified at UU where the average number of students allocated to a final year project supervisor in Biomedical Sciences per year has risen from two to seven in the last decade. Although supervisors get a small stipend of money to purchase consumables for the practical delivery of these projects, this is largely insufficient and is normally supplemented by other financial resources. However, this approach is increasingly unsustainable as student numbers increase, so dry-lab projects are clearly more financially viable, especially as student numbers are only likely to increase further in coming years.

Furthermore, increased student recruitment to our distance-learning courses in Biomedical Science at UU means that provision of dry-lab projects will become more commonplace. This is important for UU’s widening participation civic agenda because the importance of the “remote laboratory” in STEM-related education has been identified as a key resource in promoting internationalization, as well as access to education for traditionally underrepresented groups [9]. Therefore, the onus is on the School to explore innovative ways of online research project provision which can be delivered remotely and still meet the learning objectives of our courses.

Finally, in terms of workload and efficiency, it is increasingly difficult for supervisors to manage bigger numbers of students working in the laboratory, both in terms of space and time. Providing a dry-lab project workflow will allow students to work through project activities in a virtual environment in their own time without the need for a supervisor in attendance and without having to access to laboratories at scheduled times. In effect, this frees up both supervisor and student from a limiting timetable where face-to-face meetings are dependent on access to laboratory facilities. Instead, a combination of synchronous IT communication and online tutorials can be used to meet, brainstorm, set tasks and review performance. Employed properly, the dry-lab project can therefore be more efficient in terms of organisation, time-commitment and availability of those involved [8].

However, we also know that increasing workloads and pressures within academia mean that many lecturers do not have the time or freedom to implement new learning techniques in the classroom [37]. Without the time to reflect on and enhance teaching practice, adoption of new approaches will always remain a challenge unless they clearly demonstrate how it will reduce workload. Therefore, dissemination of this case study may help to persuade educators how a dry-lab project can actually solve many issues at once.

### Limitations

However, it is important to consider potential limitations to the work which could be addressed in future evaluations of this type. The small number of participants in the pilot group may have meant some important themes were not considered. A larger pilot group, ideally spread across several different projects, would have been more holistic and would help avoid bias that may come with one practitioner. Ideally, it would be best to randomly allocate students to wet- or dry-lab projects and compare their experiences. One study made students take both wet- and dry-lab experimentation and compared their experiences, which helped students develop more appreciation of scientific practice [3]. However, even this approach acknowledged some hard-to-control variables, such as personal circumstances and supervisor input. It also removes the idea of choice from students, which runs contrary to the wishes of students as shown in the data above. Nevertheless, more projects like this are required to robustly compare the wet- and dry-lab experience for students.

The survey carried out here was necessarily student-centred, but it would have been advantageous to do a staff-focused survey as well to canvas their experience of delivering a dry-lab project. It may well be that the same problems experienced by students in the mode of learning would be manifested in staff. Notably, some staff have also provided anecdotal evidence of difficulties with motivation, engagement and technology, so these are not student-specific issues. Indeed, it is worth noting that during the COVID-19 pandemic, staff were probably more likely to have caring responsibilities (e.g., home-schooling) than students, on top of dealing with a dramatically changed role in teaching and research as work moved off-campus. Staff wellbeing is also important to consider as a factor which may have contributed to the staff-student interactions which have been highlighted above as so important to the overall student experience of dry-lab projects. Capturing the staff experience of this entire process would provide a useful comparator for the student results reported here. Unsurprisingly, others have also seen the pandemic as a possible catalyst for change and have engaged with staff across various universities to put aside their preconceived ideas on research projects and work collaboratively to share ideas and create outputs [38–40]. This has led to a suite of open-access resources being made available to help staff develop, manage and deliver non-traditional projects. The need for this is clear as the authors conclude; *“We cannot return to our old ways – the worlds of work and education have changed forever.”* Interestingly, the results from this project corroborates evidence from a previous survey, collected from Level 5 and 6 students across 16 Universities in the UK, which concluded there was a need for the sector to re-think its provision of undergraduate projects, and the range of projects offered, in order to address student needs and career aspirations [29].

Looking to the future, it would be interesting to follow this cohort of students to track their employability statistics and the types of job they progress to. This might tell us if the lack of practical laboratory skills is a barrier to gaining employment in the Life Sciences sector. Alternatively, it may transpire that the gain in digital skills may well prove to be an advantage which employers valued even more highly following the experiences of the COVID-19 pandemic. A follow-up survey of these student respondents in this project in one or 2 years could be very illuminating.

## Recommendations

**FIGURE 13:**
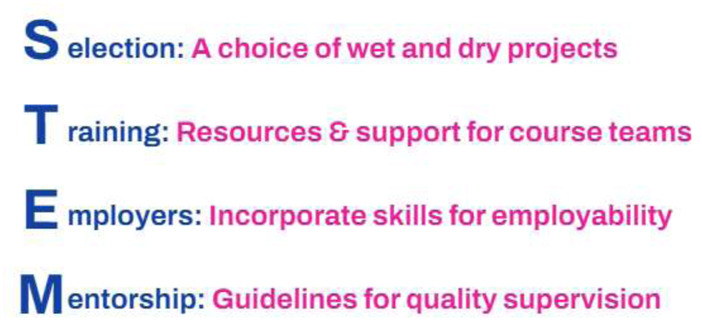
Recommendations for enhancing final year project provision.

The ideas, opinions and themes discussed above can be summarised in the following recommendations, based on the appropriate acronym ‘STEM’ (Figure 13).

### Selection

Providing choice in the types of project available is key to empowering students to choose a final year project that suits them best. It is highly recommended that bioscience courses offer a variety of dry-lab and wet-lab projects as this provides more choice for students to play a proactive role in tailoring their final year experience to suit their individual circumstances, strengths and future career aspirations. Ideally, projects should be a hybrid design, allowing students to gather both wet- and dry-lab skills [3].

### Training

Training and continual learning is essential for staff to develop the necessary skillsets required to deliver dry-lab projects effectively. Course teams are encouraged to nominate a coordinator who can monitor and disseminate the ever-growing number of resources that can be used to facilitate dry-lab project provision. These include digital tools, case studies, ‘off-the-shelf’ projects, design-your-own-project toolkits and open-access datasets. However, many colleagues are not aware of these and require direction on where to find them and how to use them. Training should be facilitated alongside these resources to inspire and encourage staff to innovate in terms of providing new types of projects. This training can then be paid forward to students undertaking the project. In our School this coordinator role is being assumed by a local Active Learning Champion.

### Employers

Regular engagement with prospective employers is important to identify the skills that they value in graduates. Course teams should utilise employer advisory board (EAB) partnerships and other industry networks to keep abreast of new skills required in the fast-changing Life Sciences sector and beyond. This information can inform the design of new projects, including those which foster dry-lab scientific skills for the world of work [39]. Indeed, some employers may even be willing to provide placement-type opportunities for student to complete final year projects in the workplace. Crucially, it needs to be articulated clearly to students which skills they will get an opportunity to develop, both to aid in their choice of project, but also so they can evidence these skills when they progress to job-seeking.

### Mentorship

Engaged supervisors are critical to a good project experience for students. Therefore, supervisors offering dry-lab projects must be aware of the need for regular communication aligned with this type of project. At the very least, it is recommended that this should include a good balance of synchronous and asynchronous interaction, with a clearly outlined schedule to guide progress. Moreover, the expectations of both student and supervisor must be established and agreed upon at the start of the project, so that there is clear understanding of the mentorship relationship and the responsibilities on both sides [36]. This is especially important for dry-lab projects where students are working remotely. This training already exists at UU for PhD supervisors, so this could easily be adapted for undergraduate project mentors.

## Conclusion

A combination of educational, financial and societal driving factors means that final year project-based learning practices in the School of Biomedical Science course need a significant change if we are to create a sustainable model of final year research project provision for future cohorts of students. In this project, evidence is presented to demonstrate that dry-lab projects can deliver an equitable, feasible alternative to wet-lab projects for students. Increased adoption of dry-lab projects can address the various pressures involved with project provision to an increasingly diverse undergraduate population in ways that can empower both staff and students alike. However, staff who are not familiar with dry-lab projects need to be motivated and supported to embed this practice routinely. In future, providing a choice of both dry-lab and wet-lab projects is highly recommended as it provides more choice for students to tailor their final year project experience to their individual circumstances, skill requirements and future career aspirations.

## Summary Table

### What is Known About This Subject?

• Non-laboratory based research projects in Biomedical Science courses are becoming increasingly commonplace in higher education.
• There is some evidence that students can benefit equally from these “dry-lab” science projects compared to traditional “wet-lab” projects.
• However, further evaluation is required to change preconceptions and expectations about dry-lab projects amongst both students and educators.

### What This Paper Adds

• This research carried out an evaluation of dry-lab project provision for students in the School of Biomedical Sciences at Ulster University.
• This research provides evidence that dry-lab project provision can be a suitable and equitable alternative for wet-lab projects.
• However, supervisors need relevant training to ensure dry-lab project provision is appropriately designed, delivered and supported.

## Concluding Statement

This work represents an advance in biomedical science because non-laboratory based research projects are increasingly commonplace, so this study demonstrates their value and provides recommendations for their implementation.

## Data Availability

The data presented in this article are not readily available per ethics approval. Further enquiries should be directed to the author.
